# Supplementary material for: Platelet Proteome Reveals Novel Targets for Hypercoagulation in Pseudoexfoliation Syndrome
Source: Int J Mol Sci. 2024 Jan 24;25(3):1403. doi: 10.3390/ijms25031403 (PMC10855978; doi:10.3390/ijms25031403)
Supplement: Supplementary file 1 [file ijms-25-01403-s001.zip › ijms-2721930-supplementary.pdf]

Table S1. ROTEM parameters for healthy controls, PEX and RVO patients in Extem, Intem and Fibtem test results.

	Healthy Controls<br>n=42	PEX<br>n=29	RVO<br>n=6
**EXTEM**			
CT (s)	59.45±9.185	58.79±6.884	60.33±4.926
A10 (mm)	57.39±4.577	60.48±5.207*	61.17±4.446
A20 (mm)	63.84±3.942	65.93±4.527	67.17±3.71
CFT (s)	79.21±17.13	69.07±15.08*	64.17±11.32*
MCF (mm)	65.03±3.81	66.97±4.492	69±2.757
Alfa (°)	74.37±3.436	76.14±3.079	77.33±2.338
**INTEM**			
CT (s)	182.2±32.17	178.4±33.16	201.2±50.82
A10 (mm)	55.74±4.283	57.72±4.052	57.5±1.732
A20 (mm)	61.64±4.035	63.21±3.986	63.83±2.639
CFT (s)	79.05±17.3	73.52±14.1	73.33±11.13
MCF (mm)	62.38±3.994	64.03±3.959	64.83±2.229
Alfa (°)	74.45±3.394	75.28±2.685	56.83±3.312**
**FIBTEM**			
CT (s)	57.82±6.79	56.68±6.171	59.67±6.154
CFT (s)	399.3±319.3	526.3±346.6	401±358.2
A10 (mm)	18.29±3.872	19.21±3.675	20.17±4.579
A20 (mm)	19.17±4.018	19.21±3.675	21.17±4.792
MCF (mm)	19.29±3.996	21.93±6.892	21.33±4.761
Alfa (°)	71.85±4.638	72.96±3.405	73.33±4.803

*$p<0.05$, **$p<0.0001$

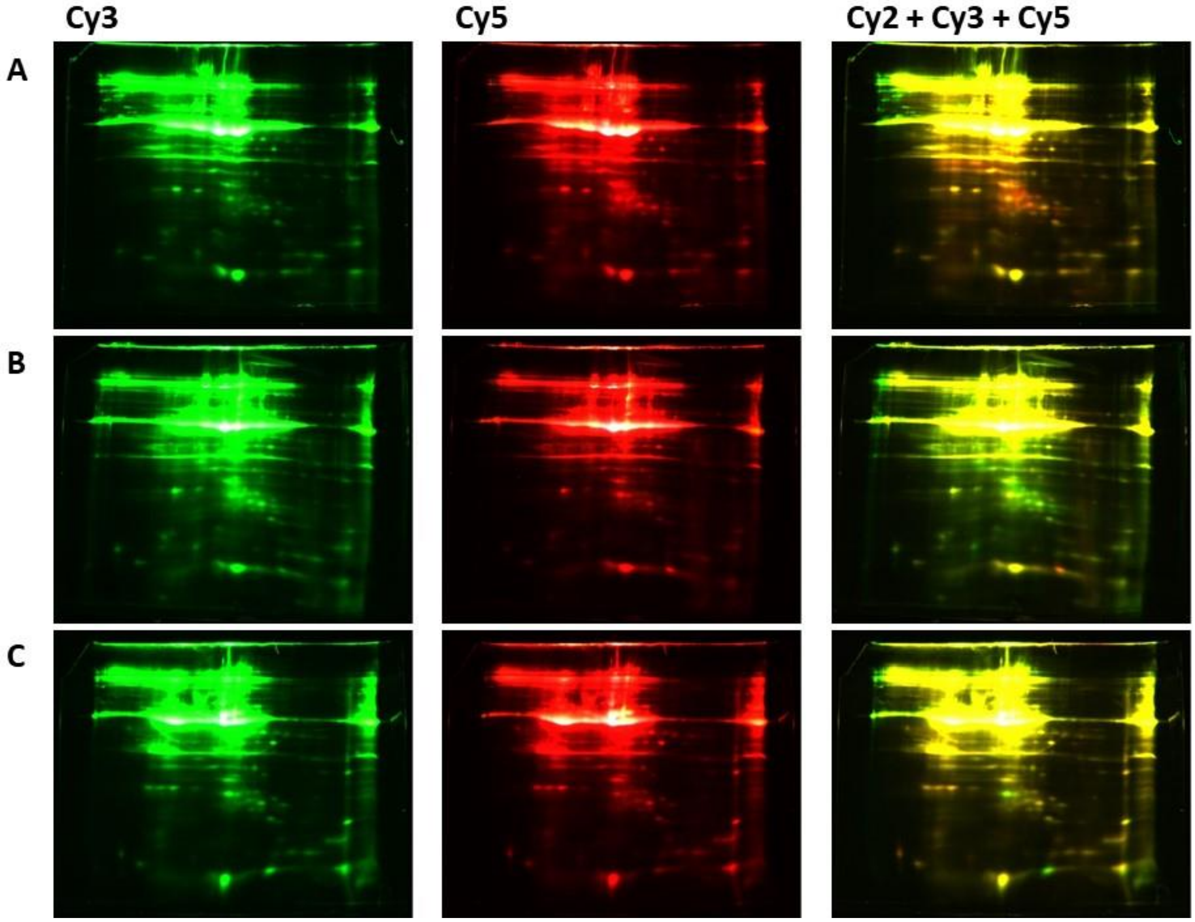

Figure S1. The gel images of 2-D Fluorescence Difference Gel Electrophoresis. An internal standard is comprised of the equal amounts of the proteins from each study group (Controls, PEX patients, and RVO patients) is labeled with Cy2. A. Protein pool of PEX patients and the controls are labeled with Cy3 and Cy5, respectively. B. Protein pool of PEX and RVO patients are labeled with Cy3 and Cy5, respectively. C. Protein pool of RVO patients and the controls are labeled with Cy3 and Cy5, respectively. The internal standard is loaded in each 2D gel experiment. Cy2+Cy3+Cy5 on the right panel of each 2D gel experiment is the merged image of Cy3 and Cy5 labeled study group and Cy2 labeled reference pool.

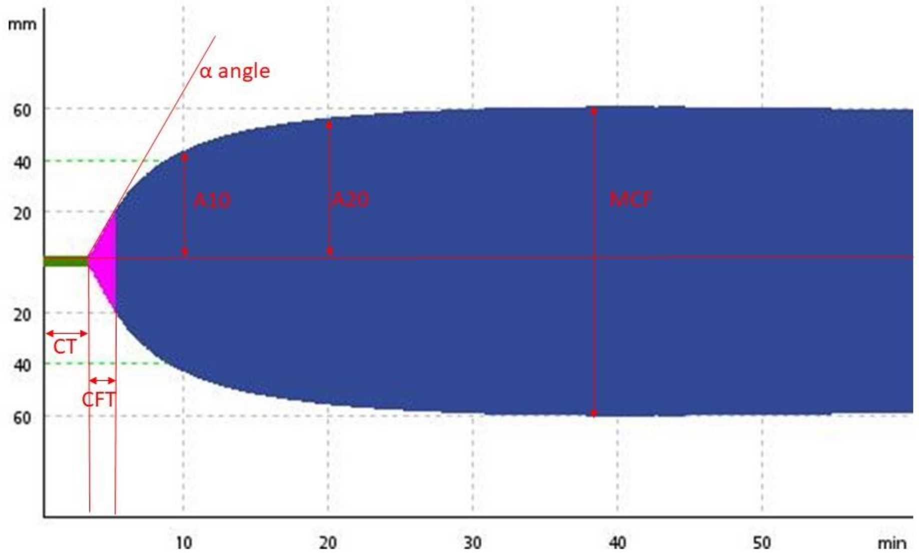

Figure S2. ROTEM parameters depicted on a EXTEM graph.

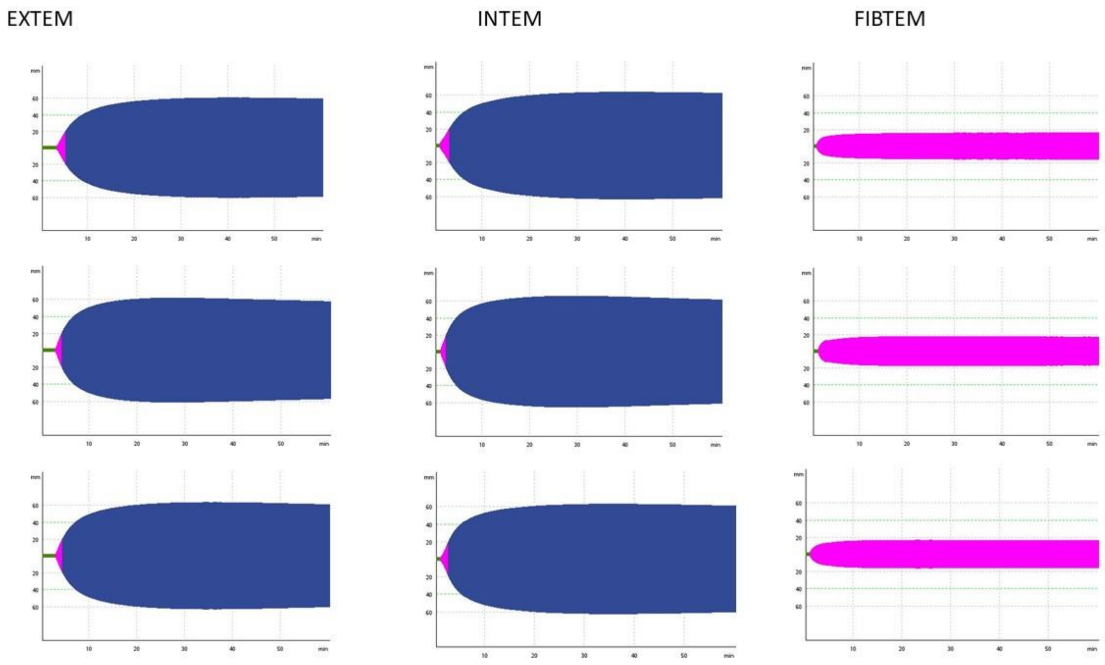

Figure S3. ROTEM tracings of 3 representative control subjects.

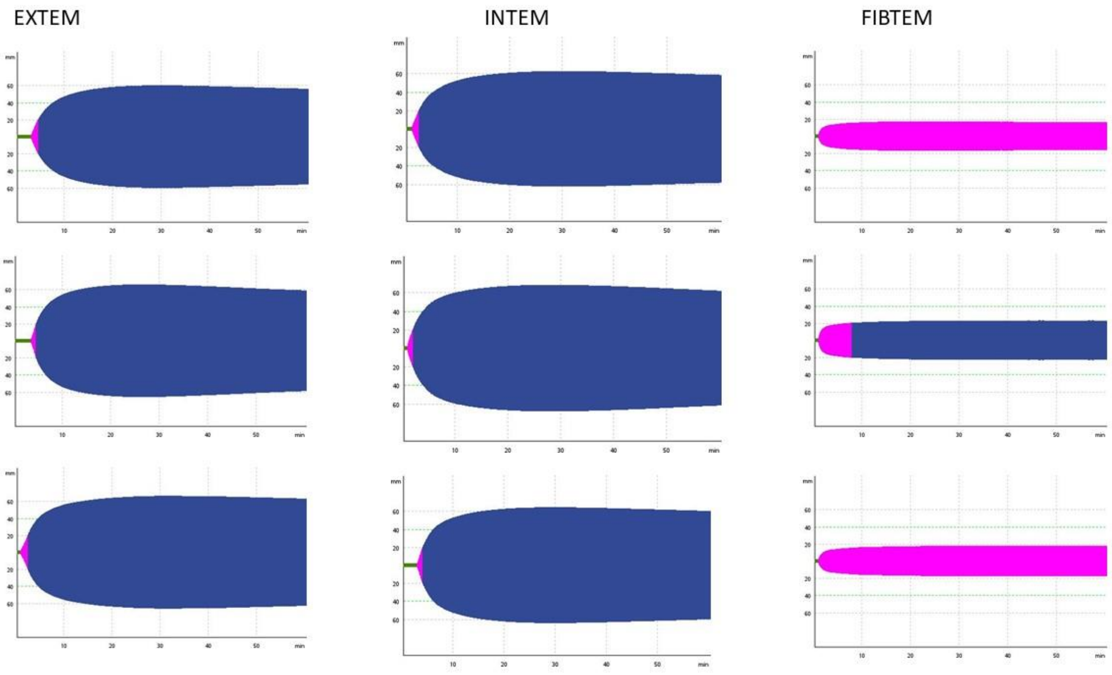

Figure S4. ROTEM tracings of 3 representative PEX patients.

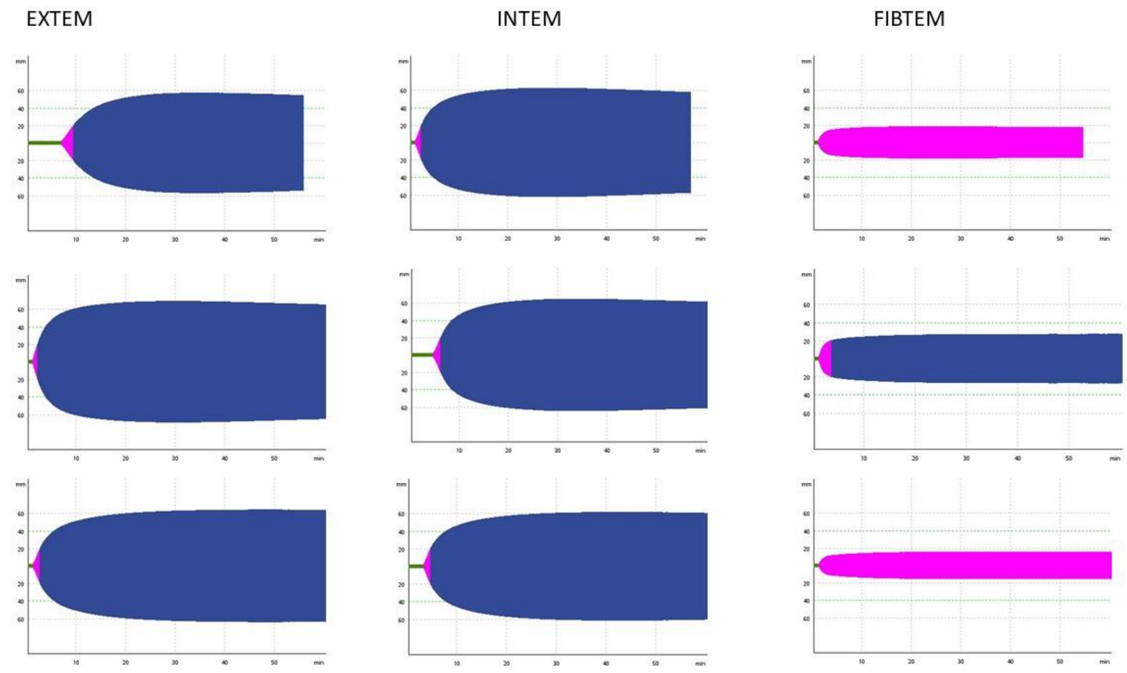

Figure S5. ROTEM tracings of 3 representative RVO patients.
